# Newly designed anterolateral and posterolateral locking anatomic plates for lateral tibial plateau fractures: a finite element study

**DOI:** 10.1186/s13018-017-0531-1

**Published:** 2017-02-23

**Authors:** Pengbo Chen, Hua Lu, Hao Shen, Wei Wang, Binbin Ni, Jishizhan Chen

**Affiliations:** Department of Orthopedics, Xinhua Hospital affiliated to Shanghai Jiaotong University School of Medicine, No. 1665 Kongjiang Road Yangpu District, Shanghai, 200092 China

**Keywords:** Locking anatomic plate, Finite element, Equivalent map, Relative displacement

## Abstract

**Background:**

Lateral column tibial plateau fracture fixation with a locking screw plate has higher mechanical stability than other fixation methods. The objectives of the present study were to introduce two newly designed locking anatomic plates for lateral tibial plateau fracture and to demonstrate their characteristics of the fixation complexes under the axial loads.

**Methods:**

Three different 3D finite element models of the lateral tibial plateau fracture with the bone plates were created. Various axial forces (100, 500, 1000, and 1500 N) were applied to simulate the axial compressive load on an adult knee during daily life. The equivalent maps of displacement and stress were output, and relative displacement was calculated along the fracture lines.

**Results:**

The displacement and stresses in the fixation complexes increased with the axial force. The equivalent displacement or stress map of each fixation under different axial forces showed similar distributing characteristics. The motion characteristics of the three models differed, and the max-shear stress of trabecula increased with the axial load.

**Conclusions:**

These two novel plates could fix lateral tibial plateau fractures involving anterolateral and posterolateral fragments. Motions after open reduction and stable internal fixation should be advised to decrease the risk of trabecular microfracture. The relative displacement of the posterolateral fragments is different when using anterolateral plate and posterolateral plate, which should be considered in choosing the implants for different posterolateral plateau fractures.

## Background

Tibial plateau fractures are common injuries affecting the lower extremities and compose 1% of all fractures [1–3]. Inadequate treatment of these fractures may result in joint instability and decrease in range of motion (RoM). Several studies have shown that open reduction and stable internal fixation (ORIF) of displaced tibial plateau fractures may ensure a more anatomic restoration of the joint surface to allow early motion without loss of reduction [1, 4–6]. Due to the specific geometry of the knee and the biomechanics of tibiofemoral joint, more than 60% of the tibial plateau fractures affect its lateral column [7, 8]. Lateral column fracture fixation with a locking screw plate has shown a higher mechanical stability than other fixation methods [7]. Meanwhile, researchers put forward that posterolateral column should be considered individually [9, 10].

**Fig. 1:**
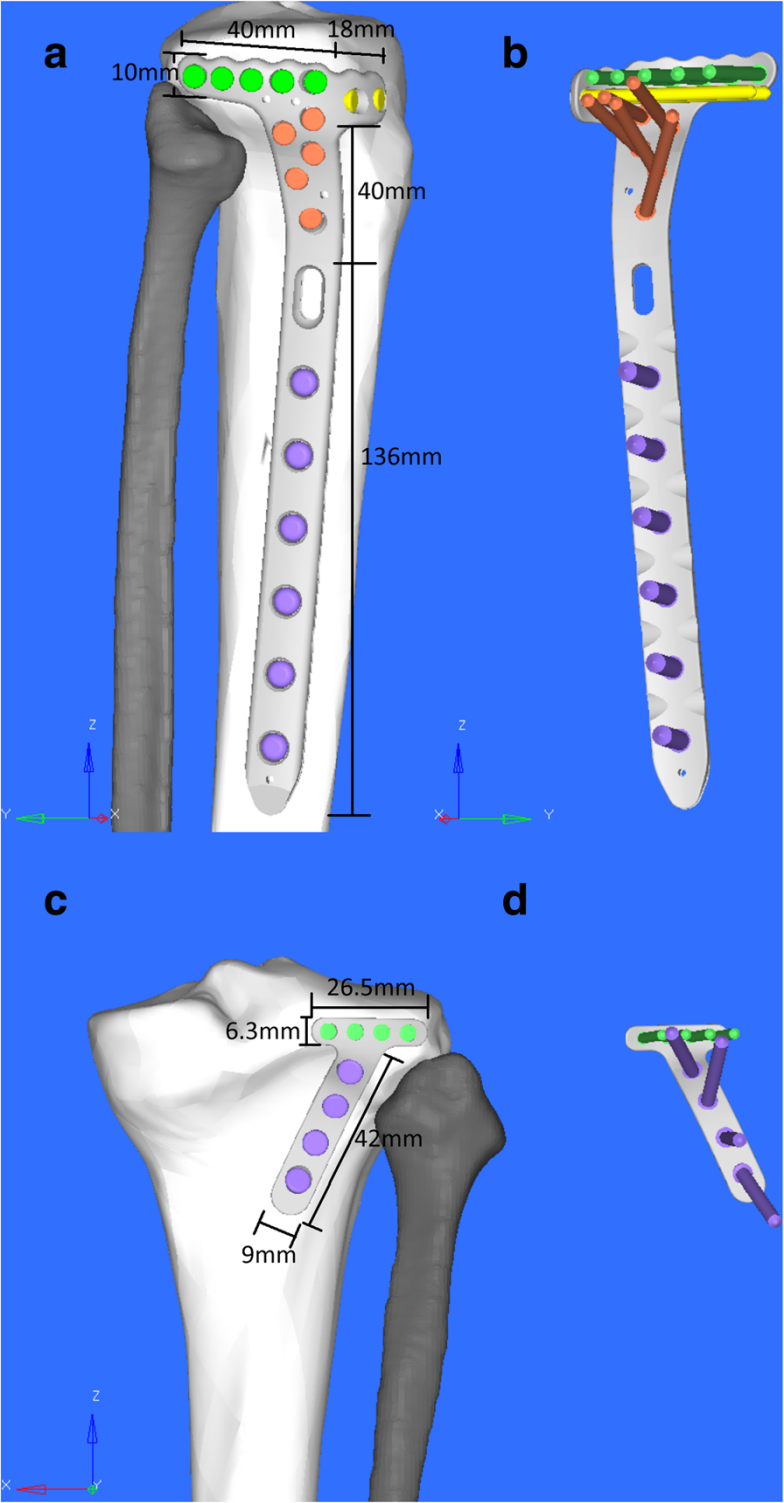
Brief introduction of the plates **a**, **b**: ALP: T shape, proximal: five raft locking screws (3.5 mm) and two crossed locking screws (3.5 mm), distal: six locking screws (5.0 mm), connection part: five locking screws (3.5 mm) with different orientation. **c**, **d**: PLP: inclined T shape with an angle of 66°, proximal: four locking screws (2.7 mm), distal: four locking screws (3.5 mm)

Complications are inevitable for some patients with lateral column tibial plateau fracture undergoing surgery [1, 11]. It is of clinical significance to investigate how to reduce complication and improve mechanical stability. It is reported that a raft of four 3.5-mm cortical screws is biomechanically stronger than two 6.5-mm cancellous screws in resisting axial compression [12]. It is also suggested that the use of crossed screws may improve the fixation stability, compared with parallel screws [13]. In the present study, we designed two novel locking anatomic plates for lateral tibial plateau (Fig. 1). The anterolateral plate was used for the anterolateral and the posterolateral fracture fragments, while the posterolateral plate was only used for posterolateral fracture fragments.

Finite element analysis (FEA) is one of the computational methods that have received wide acceptance in the field of orthopedic research, which would allow not only detailed quantitative estimations of displacement but also the load distributions in both the simulated surgical implants and the surrounding bones [14]. FEA has been shown to potentially stand for good predictors of bone fracture [7, 15, 16]. In the present study, FEA was employed to investigate if the novel plates can provide sufficient fixation strength for lateral tibial plateau fracture and the respective characteristics of the fixation complexes of posterolateral tibial plateau fractures when these novel plates were used.

## Methods

A three-dimensional (3D) tibia finite element model was constructed based on computed tomography (CT) scan data of a healthy adult man, who was 33 years old, 170 cm in height, and 60 kg in body weight. Initial CT data of the tibia was obtained with 1-mm cuts from his right leg. The 3D model of the tibia was constructed from the CT data in the Digital Imaging and Communications in Medicine (DICOM) format using Mimics software (v16.0, Materialize Company, Leuven, Belgium) and then imported into Geomagic Studio Software (v2014, 3D system Inc., Rock Hill, SC, USA) for smoothing and polishing the surface. The STEP format of the 3D tibia model was saved. All the 3D models of the screws and plates were created using computer-aided design software with the characteristics shown in Fig. 1.

**Fig. 2:**
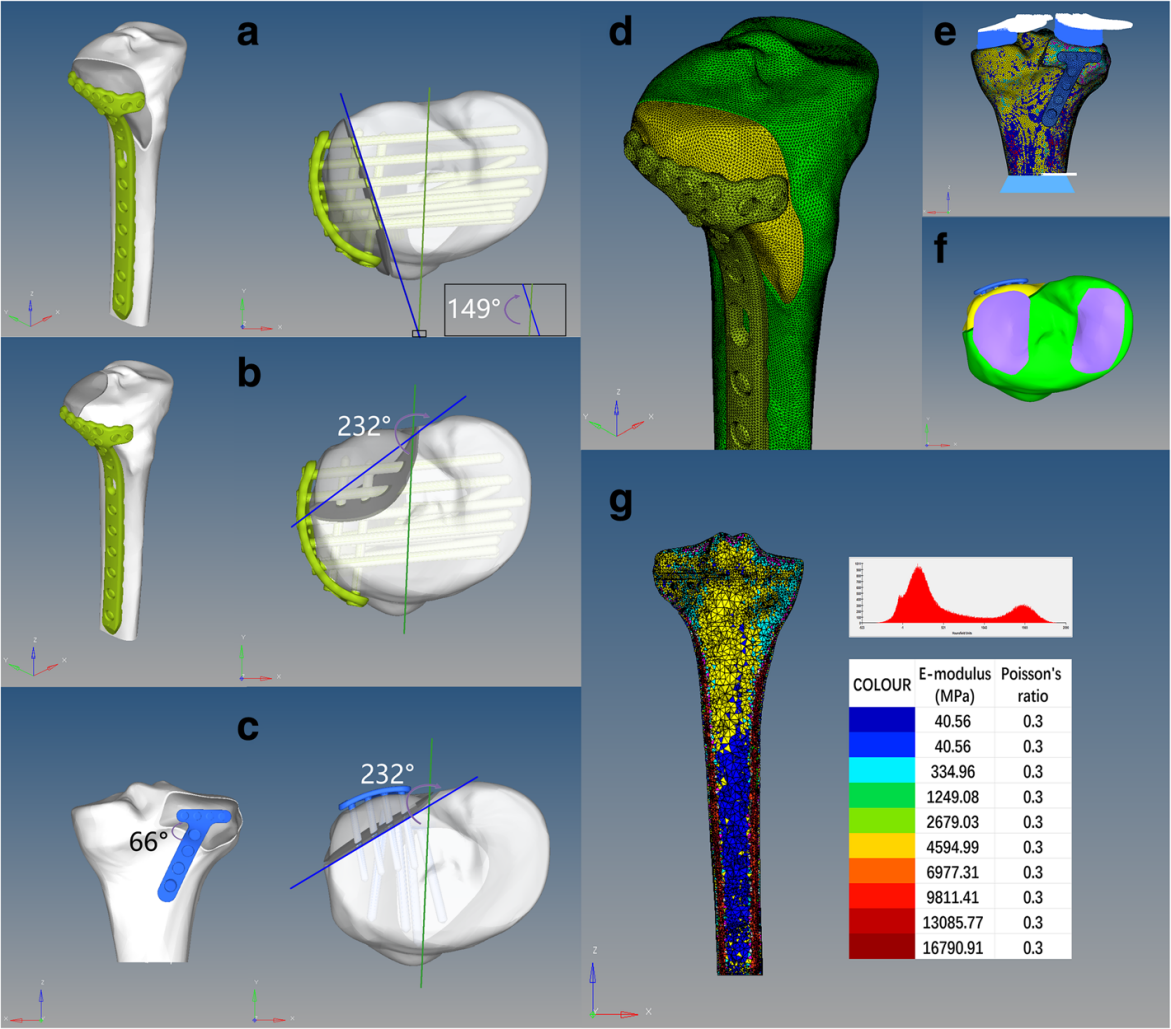
Brief introduction of the fracture models and the FE models. **a** SALF + ALP. **b** SPLF + ALP. **c** SPLF + PLP. **d** The FE model of SALF + ALP after meshing. **e** The FE model of SPLF + PLP with axial stress and constrain. **f** The location of axial stress. **g** A section of FE model after assigning the materials, the distribution of CT value, and the scale of materials. FLA: the green line connects the middle point of the posterior cruciate ligament’s insertion on the tibial plateau, and the medial 1/3 point of the tibial tuberosity was acted as a neutral axis; the blue line connects the two sides of fracture line

The 3D models of tibia and plate-screw system were then imported into Hypermesh software (v13.0, Altair Engineering Inc., Michigan, USA). The fracture models were created according to our preliminary study with certain fracture line angle (Fig. 2). The plate-screw systems were then placed in the right place simulating fracture fixation models. There were three models for the fracture fixations: single anterolateral plateau fracture with anterolateral plate (SALF + ALP, Fig. 2a), single posterolateral plateau fracture with anterolateral plate (SPLF + ALP, Fig. 2b), and single posterolateral plateau fracture with posterolateral plate (SPLF + PLP, Fig. 1c). Meshing and subsequent establishment of the finite element model were also performed with this software. Tetrahedral ten-node elements (C3D10M) were used to mesh all parts of the FE models; the nodes and elements information are summarized in Table 1.

**Table 1:** Parameters of the FE models

Nodes/elements of	SALF + ALP	SPLF + ALP	SPLF + PLP	Nodes/elements of
Plate	88505/51456	88598/51534	12252/6388	Plate
Screws	104574/56866	105052/57238	23983/12455	Screws
Fragment	91983/58212	76939/49743	33405/20754	Fragment
Tibia shaft	829695/558153	837173/562653	466951/314994	Tibia shaft

The materials of the plate-screw system were assumed to be homogeneous, isotropic, and linear elastic [7, 17]. The material properties of the plate-screw system (titanium alloy) were assigned according to the manufacturer specifications and previous studies with an elastic modulus of 78000 MPa and a Poisson ratio of 0.3 [18], while the tibia was assigned by a novel method in Mimics after meshing in Hypermesh. Average CT values of each tibia element were calculated by Mimics automatically with a corresponding elastic modulus shown in Fig. 2g and a Poisson ratio of 0.3. In that way, we did not need to distinguish the boundary of cortical and cancellous bones artificially.

The contact surfaces between the plates and screws were assumed as sharing the common nodes to simulate the locking screws so were the contact surfaces between the screws and the bone [19]. For the contact surfaces between the fragments, it was assumed with a frictional coefficient of 0.4 [20]. Axial forces of 100, 500, 1000, and 1500 N with a distribution of 60% to the medial compartment were applied to simulate the axial compressive load on an adult knee [17, 21, 22] (Fig. 2e, f). The distal part of the tibia was constrained without displacement (Fig. 2e).

Subsequently, the finite element models were imported to Optistruct software (v13.0, Altair Engineering Inc., Troy, MI, USA) and performed the analysis process. In our study, the equivalent maps of displacement and stress of the fracture fixation models were output.

Relative displacement (RD) was calculated along the fracture lines (the displacement of the triangular fragment side minus the shaft side). Displacement of different axes had an orientation. The positive directions of Z, Y, and X axes were from distal to proximal, anterior to posterior, and right to left.

## Results

### Displacement of models

**Fig. 3:**
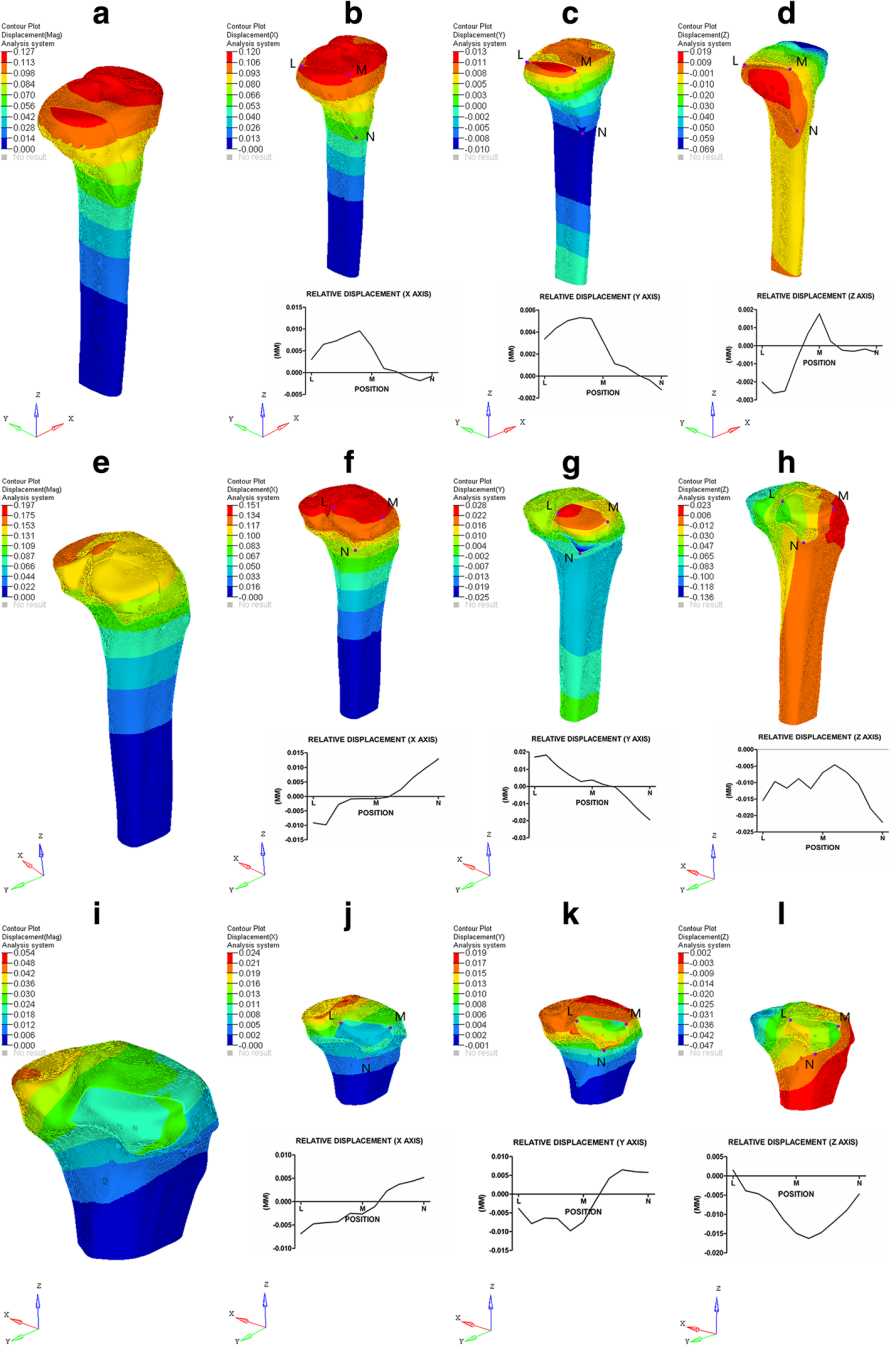
The results of displacement under 500 N axial stress. Total (**a**), X axis (**b**), Y axis (**c**), and Z axis (**d**) displacement and their RD curves of SALF + ALP; total (**e**), X axis (**f**), Y axis (**g**), and Z axis (**h**) displacement and their RD curves of SPLF + ALP; total (**i**), X axis (**j**), Y axis (**k**), and Z axis (**l**) displacement and their RDcurves of SPLF + PLP. Point L and M are two boundary points of tibia plateau along the fracture lines and point N is most distal point of the fracture fragments

The maximal displacement values of the three fixations under different loads are shown in Table 2. The displacement of each complex increased parallelly with the loads; the same was true for the equivalent maps; therefore, only the equivalent displacement maps of 500 N for each fixation are displayed in Fig. 3. RD along the fracture lines from L to M then to N are plotted as curves which revealed the details of the fracture fragments’ movements (Fig. 3b–d, f–h, j–l). L and M were two border points of fracture lines at the articular surface, and N was the lowest point of the triangle fragment.

**Table 2:** Displacement values of different FE models

	SALF + ALP				SPLF + ALP				SPLF + PLP			
Max displacement (mm)	100 N	500 N	1000 N	1500 N	100 N	500 N	1000 N	1500 N	100 N	500 N	1000 N	1500 N
MAG	0.02531	0.12658	0.25315	0.37973	0.03936	0.19680	0.39360	0.59040	0.01078	0.05390	0.10780	0.16170
X	0.02392	0.11961	0.23922	0.35883	0.03011	0.15056	0.30112	0.45168	0.00478	0.02391	0.04782	0.07173
	−0.00008	−0.00039	−0.00078	−0.00116	−0.00009	−0.00045	−0.00089	−0.00134	−0.00004	−0.00018	−0.00036	−0.00054
Y	0.00264	0.01322	0.02644	0.03966	0.00559	0.02793	0.05586	0.08379	0.00384	0.01922	0.03843	0.05764
	−0.00204	−0.01019	−0.02038	−0.03057	−0.00502	−0.02512	−0.05024	−0.07536	−0.00011	−0.00056	−0.00112	−0.00168
Z	0.00482	0.02408	0.04816	0.07224	0.00590	0.02949	0.05897	0.08846	0.00046	0.00228	0.00456	0.00684
	−0.01381	−0.06907	−0.13813	−0.20720	−0.02716	−0.13578	−0.27156	−0.40734	−0.00947	−0.04734	−0.09467	−0.14200

In 3D space (X, Y, and Z axes), the fixed fragments’ displacement showed heterogeneous. The maximal displacement values of SALF + ALP under 500 N load were 0.120 mm (X axis), 0.013 mm (Y axis), −0.069 mm (Z axis), respectively. On X and Y axes, the relative displacement values from L to M were both positive numbers, while from M to N, they turned to negative (Fig. 3b, c). On Z axis, the relative displacement value was negative in most cases, except for around the M point (Fig. 3d).

The maximal displacement values of SPLF + ALP under 500 N load were 0.151 mm (X axis), 0.028 mm (Y axis), and −0.136 mm (Y axis). On X axis, the relative displacement value decreased from L to a critical point which was just below the point M, and then the relative displacement value increased from this critical point to point N (Fig. 3f). On Y axis, the relative displacement curve reversed compared to X axis (Fig. 3g). On Z axis, the relative displacement value decreased from point L to M, and then increased gradually to the maximum at point N.

The maximal displacement values of SPLF + PLP under 500 N load were 0.024 mm (X axis), 0.019 mm (Y axis), and −0.047 mm (Z axis). On X axis, the curve of relative displacement was similar to that of SPLF + ALP (Fig. 3j); while the curve in Y axis was quite different from that of SPLF + ALP, the maximum negative relative displacement value was at the point between points L and M, and the maximum positive displacement value was between points M and N (Fig. 3k). On Z axis, the relative displacement curve displayed “V” type, and the maximum negative displacement value was close to point M (Fig. 3k).

### Stresses in the fixation complex

**Fig. 4:**
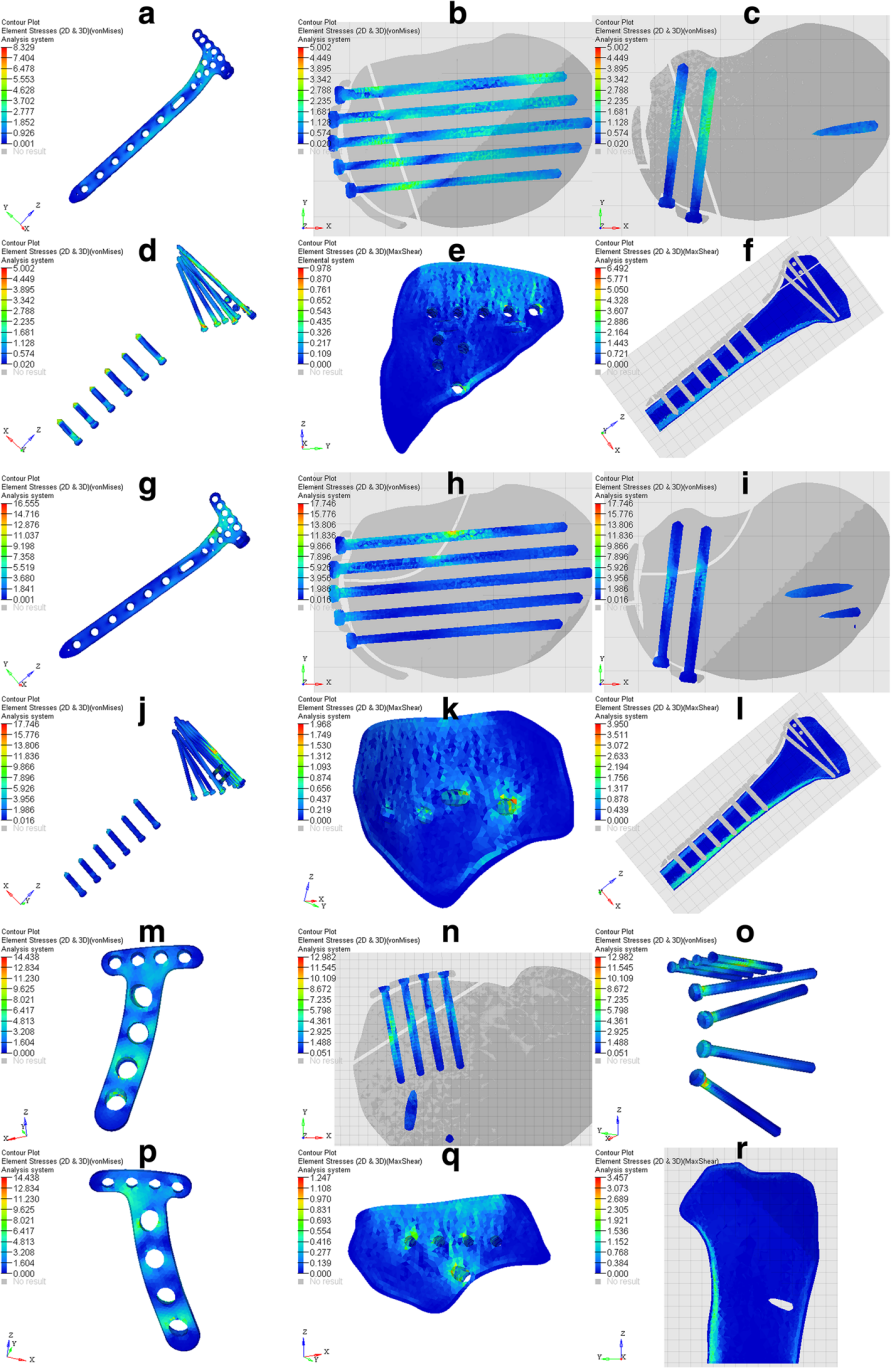
The results of stresses. **a**–**d** Plate and screws von Mises stress of SALF + ALP. **e**, **f** Bone max-shear stress of SALF + ALP. **g**–**j** Plate and screws von Mises stress of SPLF + ALP. **e**, **f** Bone Max-shear stress of SPLF + ALP. **g**–**j** Plate and screws von Mises stress of SPLF + PLP. **k**, **l** Bone Max-shear stress of SPLF + PLP. **m**–**p** plate and screws von Mises stress of SPLF + PLP. **q**, **r** bone Maxshear stress of SPLF + PLP

The stress values under different loads are summarized in Table 3. Logically, the stresses recorded in the fixation complex increased with the axial force. By comparison, the equivalent stress map of each fixation under different axial forces showed a similar distributing characteristic, and only the stress map of 500 N was presented herein. Figure 4 shows the equivalent von Mises stress of the three plate-screw systems and the max-shear stress of the bone under 500 N load. For the plates of SALF + ALP and SPLF + ALP, von Mises stress concentrated in the bent area just below the holes for raft screws (Fig. 4a, d, g, j). The stress concentration was observed on the middle section of the holes for raft screws of SPLF + ALP (Fig. 3g). For the plate of SALF + ALP, von Mises stress seemed to concentrate in the area surrounding the holes (Fig. 4m, o, p). For the screws of all the three fixations, the stresses were concentrated surrounding the fracture lines on the screws (Fig. 4b, c, h, i, n). The maximum max-shear stresses of the fracture fragments were found at the screw holes near the fracture surfaces (Fig. 4e, k, q). For the tibia shaft, stress transmitted mostly by cortical bones, especially the medial and posterior cortical bones (Fig. 4f, l, r).

**Table 3:** Values of stress of different FE models

SALF + ALP					SPLF + ALP				SPLF + PLP			
Max von Mises stress (MPa)												
Plate	1.666	8.329	16.658	24.986	3.311	16.555	33.110	49.665	2.888	14.438	28.876	43.314
Screws	1.000	5.002	10.004	15.006	3.549	17.746	35.493	53.240	2.596	12.982	25.965	38.947
Max-shear stress (MPa)												
Fragment	0.196	0.978	1.957	2.935	0.394	1.968	3.935	5.903	0.249	1.247	2.494	3.741
Tibia shaft	1.298	6.492	12.985	19.477	0.790	3.950	7.900	11.850	0.691	3.457	6.914	10.371

**Fig. 5:**
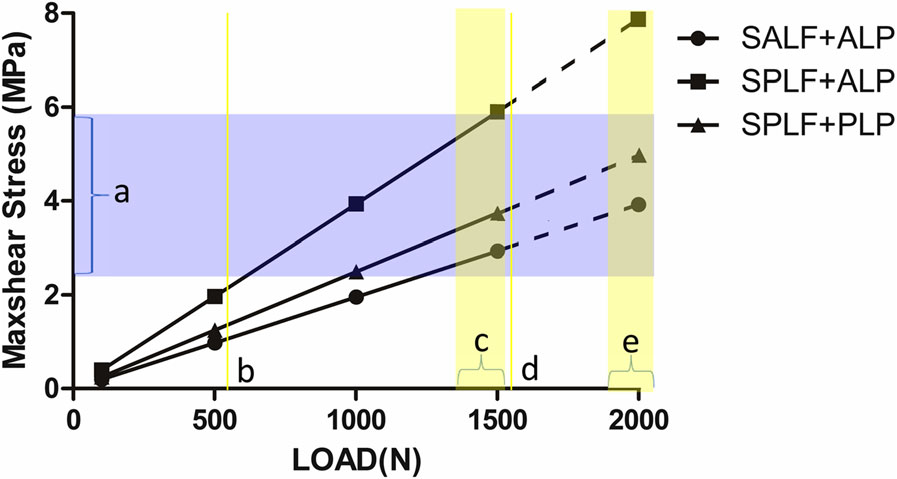
The Max-shear stress surround the screw holes and relation to the trabecular bone shear strength. **a** Trabecular bone shear strength, 2.4–5.8 MPa. **b** Two legs standing, 600 N, 100% body weight. **c** Flexion motions (bending knee, sitting down, standing up), 1320–1560 N, 220–250% body weight. **d** One leg standing, 1620 N, 270% body weight. **e** Up and down the stairs, 1860–2100 N, 310–350% body weight

The maximum max-shear stresses of the trabecular bones are summarized in Fig. 5, illustrating the possible risk of trabecular fractures for each model under different loads or motions.

## Discussion

More attention should be paid on tibial plateau fractures, especially on lateral plateau fractures, as lateral column would be affected by more than 60% of these fractures [7, 8]. Our design of two plates in the present study utilized the raft theory, resulting in a more stable tibial plateau after ORIF, compared to the normal plates. It has been demonstrated that the space between the apex of fibular head and lateral wall of plateau is sufficient for horizontal arm of the plate passing through [23]. The plates were designed as T shape. PLP was inclined with T shape, resulting in a more adequate visualization of the plate’s shaft part when screw was inserted. ALP has two backward screw holes. The two screws were crossed with the raft screws, which can be used to fix posterior plateau fragment. This structure could provide a stronger fixation [13].

In the present study, FEA was employed to demonstrate the strength of the three fixations and their characteristics under axial stresses that could not be observed by other mechanical test methods. In order to achieve an accurate outcome, a quite small size of 2D element (1 mm) was employed in these models. The nodes and element numbers of 3D elements are shown in Table 1, proving the veracity of our FE models as they were sizable. The previous methods to assign materials and properties for the cortical and trabecular bones were troublesome and imprecise as artificial or semi-artificial segmentation of different kinds of bone were required [7, 24]. However, the method utilized in the present study was a convenient way with the outcome shown in Fig. 2g. As seen numerically, we obtained a model with cortical and trabecular bones’ E-modulus similar to the research reported previously [7, 17, 25]. The cortical thickness differed from the tibial plateau to the shaft, and the trabecular bone of the plateau and tibial medullary cavity were assigned as different materials. These results showed that our FE models were more precise and closer to the reality than previous models, with a homogeneous cortical thickness and trabecular bone as a whole.

Overall, the maximum RDs achieved in the present study were far below 2 mm, which is usually considered clinically to evaluate if the reduction of a split tibial plateau fracture succeed [7, 26]. Therefore, all the three fixations developed in the present study were judged successful.

**Fig. 6:**
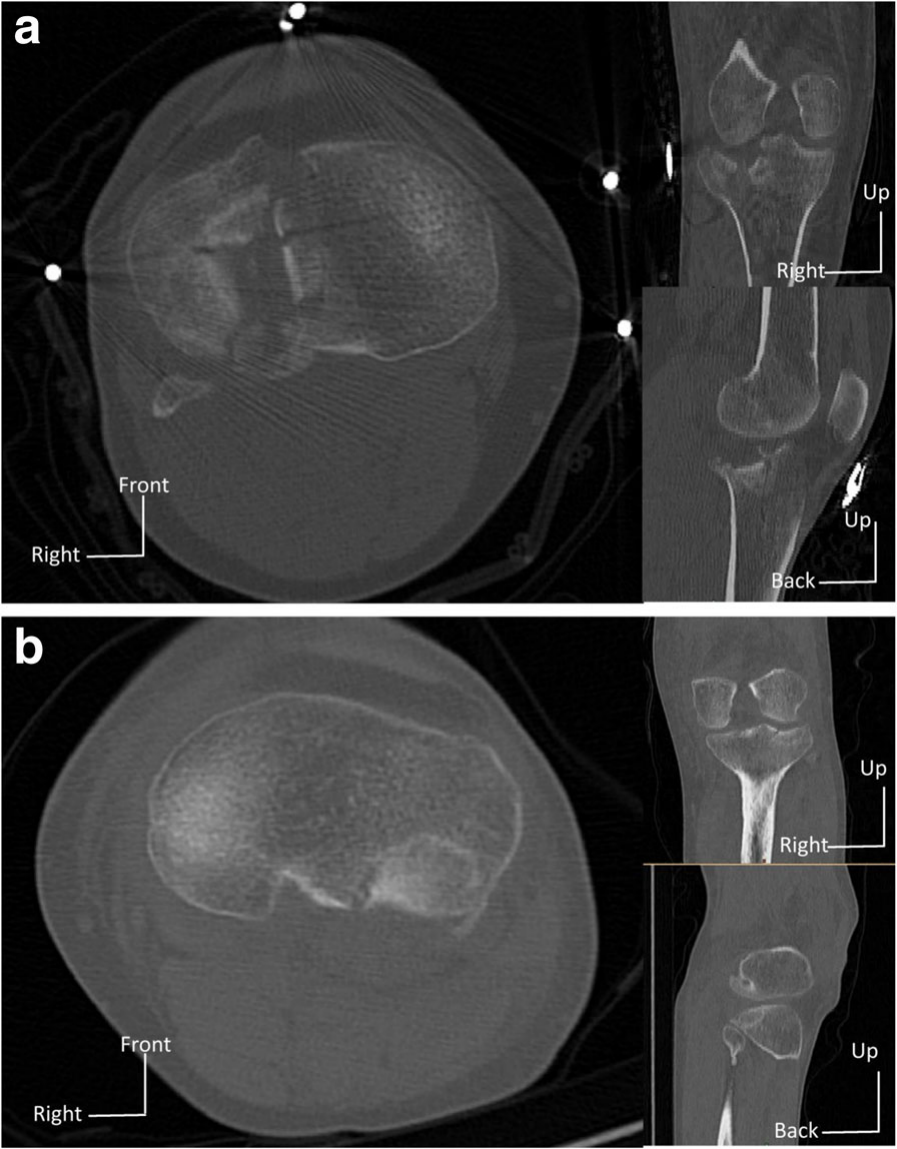
Patients with posterolateral tibial plateau fragments. **a** Posterolateral fracture with the articular surface suffering a severe collapse or syntripsis. **b** Posterolateral fracture with slight collapse or a whole fragment

For the RD value and its curves of SALF + ALP, the fracture fragment was found getting closed to the main tibia part at the articular surface height under axial stresses, while getting separated at the lower triangle of fragment. The extrusion motion is considered as a good result for fractures at the articular surface as it provides a greater possibility of absolute stability and primary bone healing [20]. However, when the articular surface suffers a severe collapse or syntripsis, the extrusion motion may bring the dislocation of the small fragments along the fracture line and therefore relative stability without extrusion motion should be considered [20]. In observations of SPLF + ALP and SPLF + PLP, although they were both posterolateral fracture, the fracture fragments moved in different ways. The triangular fragment of SPLF + ALP was more likely to separate slightly from the tibial shaft at the articular surface height, while it was adverse of SPLF + PLP. Therefore, the ALP fitted better the posterolateral fracture with a severe collapse or syntripsis at the articular surface (Fig. 6a), while PLP fitted well the posterolateral fracture with slight collapse or a whole fragment (Fig. 6b). Motions of Z axial were overall under 0.1 mm, which showed a perfect stiffness of the fixations.

On the other hand, the stresses were compared to the maximum resistance of the simulated materials. In all the tests, the maximum von Mises stress of the implants was 53.240 MPa, which was far smaller than the maximum resistance of 795 MPa (titanium alloy)[7]. No bending or mechanical damage of the screws and plates occurred.

Von Mises stress was concentrated around the connection of proximal raft screws and the distal shaft screws of ALPs so as to prevent the separation of fragment. In SPLF + ALP, the stress was concentrated on the middle section of the proximal raft screws as the plate was placed across the fracture line. The stress concentration occurred at the bottom, and cusp of the shaft screws on ALPs was caused by the stress transmitted by the two sides of cortical bone. Von Mises stress on the implants of SPLF + PLP was concentrated on the junctions of the plate and the screws with a relative homogeneous distribution on the plate. This may be caused by the stress transmitted by the lateral-side cortical bone. The stress concentrations along the fracture lines were easy to be understood.

For the bones, the max-shear stress was calculated to show the risk of trabecular microfracture. The maximum max-shear stresses of the fracture fragments were found at the screw holes near the fracture surfaces. Trabecular microfracture may bring the screw loosening, leading to the failure of ORIF. Carrera et al. have summarized that the shear strength of trabecular bone might vary from 2.4 to 5.8 MPa [7]. It has been reported that the knee joint axial stress ranged from 100 to 360% of body weight during activities of daily living [27]. According to these observations, our test load should be up to 2160 N as the body weight of this patient was 60 kg. The maximal axial load we performed in the tests was 1500 N, which might be an imperfection of our study. As shown in Fig. 4, the SPLF + ALP had the highest risk of trabecular microfracture, suggesting the patient should decrease taking the stairs and other drastic actions after ORIF. Other fixations also had the risks of trabecular microfracture, which needs further study.

## Conclusions

The two novel plates developed in the present study can fix well lateral tibial plateau fractures involving anterolateral fragment and posterolateral fragments. Motions after ORIF should be advised to decrease the risk of trabecular microfracture. The RD of the posterolateral fragments was different when using ALP and PLP, which should be considered in choosing the implants when dealing with different posterolateral plateau fractures.

## Abbreviations

ALP: Anterolateral plate
DICOM: Digital Imaging and Communications in Medicine
FEA: Finite element analysis
ORIF: Open reduction and stable internal
PLP: Posterolateral plate
RD: Relative displacement
ROM: Range of motion
SALF: Single anterolateral plateau fracture
SPLF: Single posterolateral plateau fracture
